# Infertile Individuals’ Marital Relationship Status, Happiness, and Mental Health: A Causal Model

**Published:** 2014-11-01

**Authors:** Seyed Habiballah Ahmadi Forooshany, Fariba Yazdkhasti, Saiede Safari Hajataghaie, Mohammad Hossein Nasr Esfahani

**Affiliations:** 1 Department of Psychology, University of Isfahan, Isfahan, Iran; 2 Department of Reproductive Biotechnology at Reproductive Biomedicine Research Center, Royan Institute for Biotechnology, ACECR, Isfahan, Iran; 3 Isfahan Fertility and Infertility Center, Isfahan, Iran

**Keywords:** Infertility, Marital Relationship, Happiness, Mental Health

## Abstract

**Background:**

This study examined the causal model of relation between marital relation- ship status, happiness, and mental health in infertile individuals.

**Materials and Methods:**

In this descriptive study, 155 subjects (men: 52 and women: 78), who had been visited in one of the infertility Centers, voluntarily participated in a self-evaluation. Golombok Rust Inventory of Marital Status, Oxford Happiness Ques- tionnaire, and General Health Questionnaire were used as instruments of the study. Data was analyzed by SPSS17 and Amos 5 software using descriptive statistics, independent sample t test, and path analysis.

**Results:**

Disregarding the gender factor, marital relationship status was directly related to happiness (p<0.05) and happiness was directly related to mental health, (p<0.05). Also, indirect relation between marital relationship status and mental health was significant (p<0.05). These results were confirmed in women participants but in men participants only the direct relation between happiness and mental health was significant (p<0.05).

**Conclusion:**

Based on goodness of model fit in fitness indexes, happiness had a mediator role in relation between marital relationship status and mental health in infertile individu- als disregarding the gender factor. Also, considering the gender factor, only in infertile women, marital relationship status can directly and indirectly affect happiness and mental health.

## Introduction

Infertility is defined as 1 year unprotected intercourse without pregnancy (1, 2). Although infertility pertains to physical problems, today it is considered not only as a gynecologic illness, but also as a biopsycho-social health problem (3)." For many couples, infertility is as much an emotional and spiritual crisis as it is a physical challenge "(4). Based on many psychological studies on infertility, it is clear that infertility and its treatment procedure are psychologically stressful. Based on the studies, although the majority of infertile individuals do not show overt psychiatric disorders, rates of anxiety, depressive symptoms, the low level of life satisfaction, sense of guilt and inadequacy, interpersonal problems, marital difficulties, and changing in sexual functioning were reported during or after medical treatment of infertility (5-8).

Stresses of infertility and its treatment procedure can damage the quality of relationships in infertile couples. If the couples cannot appropriately cope with these stresses, those may lead to several psychological problems for each partner that can considerably affect their marital relationship with each other. Such stressful relationships per se can intensify stresses and distresses related to infertility. Thus, in this position a vicious circle develops that can negatively affect couple’s mental health mutually. Moreover, it has been argued that the diagnosis of infertility may magnify and intensify the disappointments and conflicts that have been previously existed in couple’s relationships with each other (5).

Although mental health of couples may be damaged by the diagnosis of infertility and couples are usually confused about the ways of handling this condition, infertility per se does not necessarily damage couple’s mental health or quality of their relationship with each other (3). Even some studies show that infertile couples report more intimacy than fertile couples (9). In fact, the influence of infertility on the mental and marital relationship health of infertile couples may be variable in different couples. While in some couples infertility can destroy their marital relationships and consequently develops many stresses, some others have reported that this crisis improved their marital relationships and intimacy (5). It seems that some features of infertile couples’ relationships have important negative or positive roles on their mental health, even when they confront with infertility. The quality of infertile couples' happiness can play such a role.

Happiness, which is as essential dimension of life and related to functioning and success (10), generally is considered to comprise three main components. These components are frequency and degree of positive affect or joy; absence of negative feelings, such as depression or anxiety; and the average level of satisfaction over a period (11). Studies show that happiness not only is considered as outcome of positive events and factors, but also considered as productive of positive outcomes especially in mental health (12). Some theoretician believes that happiness is related to satisfying social and interpersonal relationships especially marital relationship (13). Based on several theories and studies, marital happiness is a significant predictor for general sense of happiness (14, 15). This relationship was even confirmed by multicultural studies (16). In fact a satisfying marital relationship by fulfilling intimacy needs of both partners enhances the rates of positive emotions between them such as happiness and consequently enhances physical and mental health of each partner (14). Studies on happiness consistently have showed a strong relationship between happiness and health, as happier people are healthier (17). This relationship has been confirmed through several studies in variety cultures and populations (18-21). Happiness and mental health are two key concepts in psychology that have considerably the overlap with each other, because both of them related to psychological well-being. However, these two concepts are regarded as two independent components in related conceptual details, mental health usually is recognized by behavioral and emotional status without any destructive dysfunctioning but happiness is usually recognized by positive and constructive emotional status (22).

Based on several studies and theoretical discussion, it is clear that there are significant relationships between marital relationship status with mental health (23-27), marital relationship status with happiness (15, 16), and happiness with mental health (19-21). But none of these studies were administrated in infertile individuals. Indeed, these studies suggest that marital relationship status and happiness are key variables to determine mental health in general population but many aspects of these relationships have remained vague so far, such as: 1. Does this relationships is significant in some special population such as infertile individuals that experience particular and different stresses and distresses compared with general population? 2. Does happiness can be considered as a mediator variable in relationship between marital relationship status and mental health in infertile individuals?

Based on several studies, that mentioned, it seems that infertile individuals may be more at risk for mental health problems, compared with fertile individuals, because they experience many social, cultural, and psychological stresses related to infertility (5). So it is necessary to identify any factors that may have a significant effect on mental health in this population. Identifying and regarding these conceptual components can be helpful to consider some critical factors in treatment procedure of infertility.

**Fig 1:**
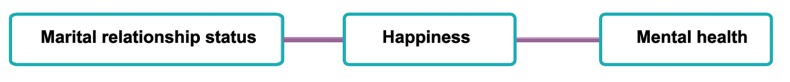
Theoretical path model for the relation between marital relationship status, happiness, and mental health.

According to our knowledge, so far no study investigated the mediator role of happiness in relationship between marital relationship status and mental health in infertile individuals through a causal model. Thus, the purpose of this study was to investigate the causal model of relation between marital relationship status, happiness, and mental health in infertile individuals. The theoretical path model for the relation between marital relationship status, happiness, and mental health is presented in the figure 1.

## Materials and Methods

The research method of this study was descriptive. The study population included all infertile men and women visited in the Isfahan Fertility and Infertility Center between August and September 2012. The sample of this study were 155 subjects (men: 52 and women: 78) whom had been visited in the Fertility and Infertility Center and selected by convenience sampling [a type of sampling in which members of the population are chosen based on their relative ease of access. This kind of sampling is common to use when applying other sampling methods accompanied by some difficulties. But in this kind of sampling there are limitations to generalization of data (28)]. Two psychologist (a man and a woman) conferred to the both men and women subjects (not with their comrades), separately in waiting room and explained the research, then if each of the subjects was volunteer to participate in the research, he/she would completed the instruments of the study (in Persian language).The Golombok Rust Inventory of Marital Status (GRIMS) is a self-report instrument contained 28 items. Each item concluded four options (0-3) based on the likert scale. This inventory was designed to measure the quality of marital relationships. It was created in 1998. The higher scores in this inventory indicate serious difficulties in marital relationships (29). The reliability of this inventory was confirmed thorough several studies (30). Psychometric components of the Persian form of this inventory were assessed by Besharat in infertile couples and its reliability was assessed as 0.92 for women and 0.94 for men (31). One item of this inventory is, for example "I am dissatisfied from our marital relationship".

The Oxford Happiness Questionnaire (OHQ) is a self-report instrument with 29 items designed to measure intensity of happiness (32). It was created in 2002 (33). Each item concluded four options (0-3), constructed to reflect incremental steps defined as: unhappy or mildly depressed, a low level of happiness, a high level of happiness, and mania. The respondents were asked to “select the one statement in each group which best describes your feeling over the past week, including today.” The higher scores in this questionnaire indicate higher levels of happiness (34). In the study of Abedi et al. (35) this questionnaire was standardized in Iranian population. Based on this study, for OHQ the reliability was assessed as 0.85, the factorial validity assessed as 0.74, and concurrent validity assessed as 0.73. One of items of this inventory is, for example "- I don’t feel happiness - I somewhat feel happiness - I feel so happiness - I am extremely happy".

The General Health Questionnaire (GHQ-28) is a self-report instrument with 28 items designed to measure mental health (36). Each item concluded four options (0-3) based on the likert scale and the higher scores in this questionnaire indicate low levels of mental health (29). In the study of Ebrahimi et al. (37) the criterion validity of this questionnaire was assessed as 0.78 and its reliability assessed as 0.97. One of items of this inventory is for example "Do you recently feel happiness in your life".

In this study, Cranach’s alpha values for the three instruments were computed. In the GRIMS, Cranach’s alpha was assessed as 0.935, in OHQ, as 0.934, and in GHQ-28, Cranach’s alpha as 0.563.

Data was analyzed by SPSS17 and Amos 5 software using descriptive statistics and path analysis.

Ethical considerations in this study concluded:

1. each of subjects who not volunteer to participate in the research was disregarded for this study.
2. The information related to each participant was secret and no organization or person with the exception of the authors reaches to these data.

## Results

Mean, standard deviation, and independent sample t test of all participants' scores in variables of the study are presented in the table 1.

According to table 1, there were no significant gender differences in marital relationship status and happiness but in mental health scores, a difference between men and women was significant. Accordingly, the levels of mental health of infertile women are significantly lower than infertile men.

To analysis of the causal model of relation between marital relationship status, happiness, and mental health, path analysis was used. The result of path analysis for all participants (including men and women) is presented in the table 2.

Based on the table 2, a direct path of marital relationship status to happiness and a direct path of happiness to mental health were significant. Marital relationship status had relatively the low direct effect on happiness but happiness had almost the high direct effect on mental health. Fitness of the theoretical presented model for all participants (including men and women) was investigated by fitness indexes (38, 39). These results are presented in the table 3.

**Fig 2:**
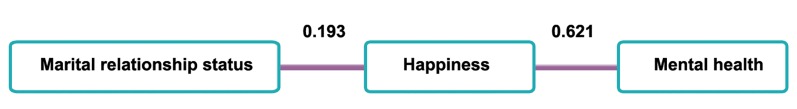
The causal model of relation between marital relationship status, happiness, and mental health in all participants (including men and women).

Based on the results of table 3, fitness of the theoretical causal model of the study was confirmed for all participants including men and women. The model with path coefficients is presented in the figure 2. Based on this model, marital relationship status had indirectly effect on mental health through happiness (indirect effect=0.119).

Also, the model of the study separately investigated men and women. The result of path analysis for women is presented in the table 4.

Based on the table 4, in women participants, the direct path of marital relationship status to happiness and the direct path of happiness to mental health were significant. Marital relationship status had relatively the low direct effect on happiness but happiness had almost the high direct effect on mental health. Fitness of the theoretical presented model for women participants was investigated by fitness indexes. These results are presented in the table 5.

**Table 1:** Mean and standard deviation of participants' scores in marital relationship status, happiness, and mental health in men and women


Variables	Mean			SD			t
	Men	Women	Total	Men	Women	Total	

**Marital relationship status**	39.192	38.307	38.689	6.048	7.014	7.046	2.077*
**Happiness**	41.788	38.243	39.712	17.200	16.081	16.560	-0.500
**Mental health**	25.038	30.995	28.386	14.982	15.125	15.273	-0.849


*; P=0.040.

**Table 2:** Standard regression weights of paths between variables in men and women


Paths		Estimate	Standard error

**Marital relationship status**	Happiness	0.193*	0.197
**Happiness**	Mental health	- 0.621 **^1^	0.059


1; Minus in this table were used because of differences between scoring the instruments, it is not indicate the negative relation between the two concepts, *; P=0.028 and **; P=0.001.

**Table 3:** Fitness indexes for the theoretical path model for the relation between marital relationship status, happiness, and mental health in all participants (including men and women).


Fitness indexes	Value	Appropriate range for fitness	Position of model	

**CMIN**	1.996*	Lack of statistical significance	Fitness	
**TLI**	0.924	>0.90	Fitness
**NFI**	0.976	>0.90	Fitness
**CFI**	0.987	>0.90	Fitness
**RMSEA**	0.080	>0.05-0.08	Fitness


*; P=0.158, CMIN; Chi-square value, TLI; Tucker lewis index, NFI; Normed fit index, CFI; Comparative fit index and RMSEA; Root mean square error of approximation.

**Table 4:** Standard regression weights of paths between variables in women


Paths		Estimate	Standard error

**Marital relationship status**	Happiness	0.254*	0.241
**Happiness**	Mental health	- 0.697**	0.072


*; P=0.026 and **; P=0.001.

**Fig 3:**
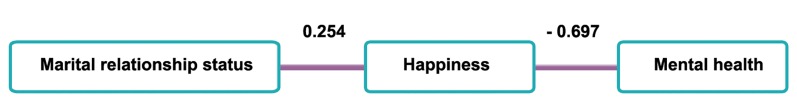
The causal model of relation between marital relationship status, happiness, and mental health.

Based on the results of the table 5, fitness of the theoretical causal model of the study in women participants was confirmed. The model with path coefficients is presented in the figure 3. Based on this model, in women participants, marital relationship status had the indirectly effect on mental health through happiness (indirect effect= 0.177). The result of path analysis for men participants is presented in the table 6.

Based on the table 6, in men participants, the direct path of marital relationship status to happiness was not significant but the direct path of happiness to mental health was significant. So, in men participants, marital relationship status had not a significant direct effect on happiness but happiness had almost the high direct effect on mental health. Fitness of the theoretical presented model for men participants was investigated by fitness indexes. These results are presented in the table 7.

**Fig 4:**
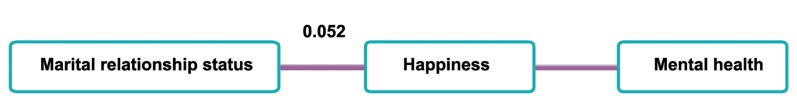
The causal model of relation between marital relationship status, happiness, and mental health in men.

Based on the results of table 7, the model of the study for men participants had a poor fitness. The model with path coefficients is presented in the figure 4. Based on this model, in men participants, the direct effect of marital relationship status on happiness and indirect effect of marital relationship status on mental health, through happiness, had not been confirmed.

**Table 5:** Fitness indexes for the theoretical path model for the relation between marital relationship status, happiness, and mental health in women


Fitness indexes	Value	Appropriate range for fitness	Position of model

**CMIN**	0.039*	Lack of statistical significance	Fitness
**TLI**	1	>0.90	Fitness
**NFI**	0.999	>0.90	Fitness
**CFI**	1	>0.90	Fitness
**RMSEA**	0.000	>0.05 - 0.08	Fitness


*; P=0.843,CMIN; Chi-square value, TLI; Tucker Lewis index, NFI; Normed fit index, CFI; Comparative fit index and RMSEA; Root mean square error of approximation.

**Table 6:** Standard regression weights of paths between variables in men


Paths		Estimate	Standard error

**Marital relationship status**	Happiness	0.052*	0.376
**Happiness**	Mental health	- 0.559**	0.098


*; P=0.700 and **; P=0.001.

**Table 7:** Fitness indexes for the theoretical path model for the relation between marital relationship status, happiness, and mental health in men


Fitness indexes	Value	Appropriate range for fitness	Position of model	

**CMIN**	3.370*	Lack of statistical significance	Fitness	
**TLI**	0.243	>0.90	Fitness
**NFI**	0.864	>0.90	Fitness
**CFI**	0.874	>0.90	Fitness
**RMSEA**	0.197	>0.05 - 0.08	Fitness


*; P=0.066, CMIN; Chi-square value, TLI; Tucker lewis index, NFI; Normed fit index, CFI; Comparative fit index and RMSEA; Root mean square error of approximation.

## Discussion

Although the main purpose of this study was not to compare infertile men and women in dependant variables of the study but the results showed significant differences in infertile men’s and women’s mental health. These findings of this study were in the line of studies showed that infertile women demonstrated more health impairment, compared with infertile men (40, 41). Also, this study showed that when participants' scores in dependent variables of the study were separated based on the gender factor, the results of the model of the study were varied for two genders. This data showed that only in women and not men marital relationship status had direct and indirect effects on mental health. Thus, these results showed that infertile women had poorer mental health compared with infertile men and marital relationship status with its effect on happiness was effective on this position of mental health in the infertile women but mental health and happiness of infertile men was not affected by their marital relationship status. It seems that some cultural factors intervene in this finding, such as high social pressures on women and extreme expectancies of them about infertility compared with men. There is the considerable evidence that infertility elicits different experience and reactions in men and women. Women tend to experience more worry about the infertility and its treatment and more tend to assume personal responsibility about this problem. Moreover, the experience of anxiety, depression, low levels of self-esteem, and psychological adjustment is more observable in women than men during infertility investigations or treatments. But it does not mean that infertile men don’t experience any stress or distress related to infertility. Historical studies on psychological effects of infertility are often concerned on women experiences of infertility, while men’s psychological stresses related to infertility have been less considered. Indeed, infertility as a crisis in couple’s life can negatively affect both partners' health and their relationships (5). This thought was confirmed by this study. Based on the results of this study, all of the scores of marital relationship status, happiness, and mental health of both men and women were not considerably appropriate as an idealistic health position. Based on the findings, both infertile men and women experience almost equal negative emotions in their personal and marital life, that indicated almost in their equal scores in marital relationship status and happiness. But it seems that for infertile women not only their marital relationship and happiness have impaired but also they experience significant mental health problematic issues that are affected by their marital relationship status with its effect on their happiness. These results indicated that infertile women experience more social and cultural stresses than infertile men about their marital role (2) while mental health and happiness of infertile men are significantly less affected by these stress factors. Some of these stresses that affect infertile women, related to sense of unsafety about stability of the marital relationship after infertility diagnosis. In many cultures divorce or even polygamy may be pursued by husband when infertility of a woman was confirmed. Although the rates of these choices are not very considerable worry about the issue can annoy the infertile women (5).

The findings of this study confirmed the causal model of relationship between marital relationship status, happiness, and mental health in infertile individuals without consideration the gender factor. This model also was confirmed in women participants but not confirmed in men participants. Based on this model in infertile women, marital relationship status directly is effective on mental health with mediation of happiness. Also the results showed that in both infertile men and women, happiness has the direct effect on mental health. The findings are the line of several studies that have showed significant relationships between these three variables (15, 16, 19, 20, 24-27) but this study was the first study that investigated these relationships through a causal model and among infertile population. There is considerable explanation for the key role of infertile couples' marital relationship status in their happiness and mental health. When a couple encounters with infertility, each of them needs to gradually experience this shocking crisis and regulates his or her emotional distress related to infertility. If the couples success to resolve these emotional conflicts, after Ahmadi Forooshany et al.short time, they can recover their individual mental health and emotional well being and then can look for appropriate solutions for treatment of infertility. But if the relationships between couples included several conflicts and dissatisfactions - especially before infertility diagnosis-, this recovery may be not acquired (5). Based on the results of this study, it seems that women more need to a satisfying and supportive marital relationship status than men to resolve the crisis of infertility that can due to their more experience of social and cultural stresses about infertility and their higher levels of affective needs in marital relationship. For both men and women, their experience of happiness significantly predicts their mental health in exposure of infertility crisis. Several studies have confirmed that positive emotions, especially happiness, have significant effects on several aspects of mental health in general population. When individuals experience happiness in long term and high levels, their personal and social functions in many fields of their everyday life improve but when they experience low levels of happiness, many aspects of their personal and social life are affected by difficulties (13).

In psychological theories and studies about close relationships, it has repeatedly reminded that happiness cannot be understood without understanding close relationships. Very studies in the field of well-being have confirmed that happy people have satisfying relationships. Based on these studies, satisfaction of marital relationships is a strong predictor of happiness (42). Even in some psychological theory such as the Choice Theory the concepts of happiness, satisfying relationships (especially in marital relationships), and mental health were considered as almost equal concepts (43). Based on the choice theory as an important and valid theory in psychology and counseling (44), happy people actually are people with satisfying relationships because based on the choice theory, basic psychological needs of human can be only satisfied in healthy and relationships. Only in this type of relationships, positive emotions, especially happiness, can be expected because positive emotions, such as happiness and pleasure, are developed when the basic physical and psychological human needs are satisfied (45). Thus, mental health is considered as the consequent of happily satisfying relationships (46). The fitness of the model of this study confirmed this psychological theoretical basis for the relationship between marital relationship status, happiness, and mental health in infertile population disregarding gender differences and also in women participants. Although this model was not confirmed in infertile men but it is necessary to investigate this model in this population with large sample size because in this study the number of men was less than women and this position may affect the results. In fact also it is reasonable that marital relationship status has more significant effect on happiness and mental health in infertile women but it seems that infertile men somewhat are affected by marital relationship status too.

## Conclusion

The findings of this study revealed the importance of attention to psychological health of infertile individuals, especially in infertile women. Also, the findings suggest that it is useful to enter some components of marital relationship status that can improve and enhance the experience of happiness in infertile couple’s relationships to mental health in these individuals. So, in the field of psychotherapy and counseling with infertile individuals probably couple therapy or couple counseling is better and more appropriate than individual therapy or counseling. Moreover, the authors suggest that psychological interventions by psychologist or counselors integrate and accompany with medical treatments presented by professional infertility centers.

To generalize these findings to Iranian population, we suggest further researches to assess the model of this study in other population of infertile couples and individuals. One of the most important limitations of this study was disregarding demographic components and personal characteristics such as highest group of age, duration of infertility, type of treatment, main reason of infertility belong to which gender. Most of these limitations were limitation of sufficient number of professional examiner to interview with subjects and invite them to participate in the study. The examiners of this research were only two psychologists, so in spite of considerable time spent for the sampling, the sample size of the study was not completely sufficient.
